# Suspected nicotine-induced acute urticaria following excessive use of smoking cessation lozenges: a case report

**DOI:** 10.3389/fmed.2026.1900012

**Published:** 2026-09-11

**Authors:** Dan Lu, Liping Chen, Chenjian Tang

**Affiliations:** 1 Department of Neonatology, Hospital of Chengdu University of Traditional Chinese Medicine, Chengdu, China; 2 Department of Geriatrics, People's North Road Community Health Service Center (The Second People's Hospital of Jinniu District), Chengdu, China; 3 Department of Acupuncture, Hospital of Chengdu University of Traditional Chinese Medicine, Chengdu, China

**Keywords:** acute urticaria, cessation, lozenges, nicotine, smoking

## Abstract

Acute urticaria is a condition characterized by the rapid onset of raised, itchy welts, often triggered by various factors. This report presents a case of acute urticaria that may be related to excessive intake of nicotine from smoking cessation lozenges. The patient had consumed a large amount of smoking cessation lozenges (containing nicotine) 3 days before the onset of symptoms, which was suspected to be caused by nicotine overdose. But, the association between nicotine overdose and acute urticaria is hypothetical and cannot be confirmed based on a single case. Following 10 days of treatment with intravenous methylprednisolone, pantoprazole, nebulized terbutaline and dexamethasone, and oral antihistamines, the patient improved significantly and was discharged.

## Introduction

Acute urticaria is defined as a rapid-onset condition characterized by the appearance of raised, itchy welts on the skin, which can be triggered by various factors, including allergens, medications, or irritants. The prevalence of acute urticaria is well-documented, the specific incidence of nicotine-related allergic reactions remains less understood, particularly concerning nicotine replacement therapies such as smoking cessation lozenges (containing nicotine). A study has found that some patients developed urticaria after using e-cigarettes, and the urticaria may be associated with e-cigarette use (1). Early research indicates that nicotine in tobacco smoke functions as an inhaled allergen, capable of triggering urticaria in sensitized individuals (2). A female smoker and cigarette factory worker developed daily urticaria triggered by tobacco exposure at work, with positive skin test reactions to several tobacco blends and complete symptom resolution during a 1-month work leave (3).

Initially, smoking cessation lozenges was used to assist smokers in quitting smoking. Research has shown that 2 milligrams of nicotine can help smokers quit smoking more effectively (4). Meanwhile, e-cigarettes could lead people to take in more nicotine than they would from regular tobacco, possibly causing a nicotine overdose. This may harm the heart and blood vessels (5). Established by Mayer in 2014, there is a disagreement between the generally accepted lethal dose and documented cases of nicotine intoxications (6). Studies have shown that nicotine toxicity presents with dyspnea, agitation, and altered mental status (7). Another case of nicotine poisoning presented with nausea, hand tremors, facial flushing, dry mouth, palpitations, vomiting, diarrhea, and confusion (8). The complexities of diagnosing urticaria can be profound, particularly when patients have initiated new medications or supplements (9, 10). The association between nicotine consumption from smoking cessation products and the onset of acute urticaria presents a noteworthy concern that necessitates further investigation.

## Case presentation

**Figure 1:**
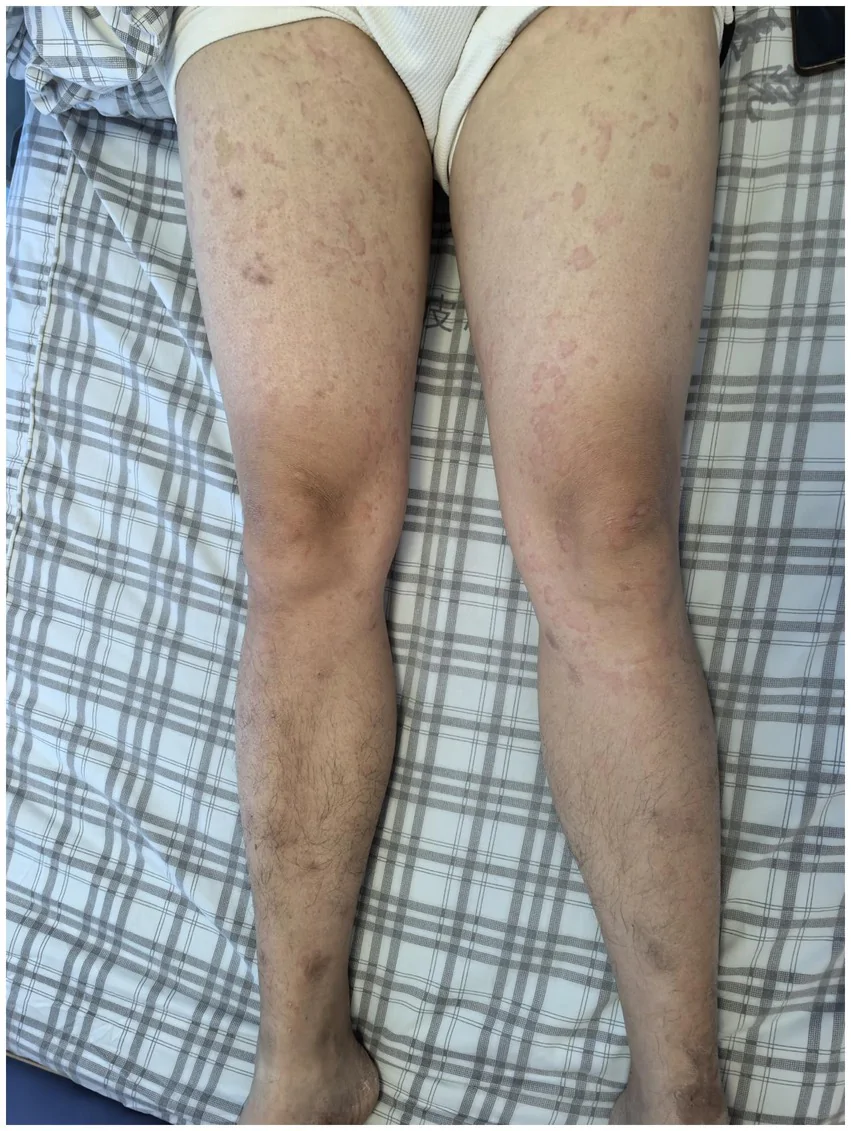
Clinical presentation of skin lesions (both lower extremities).

**Figure 2:**
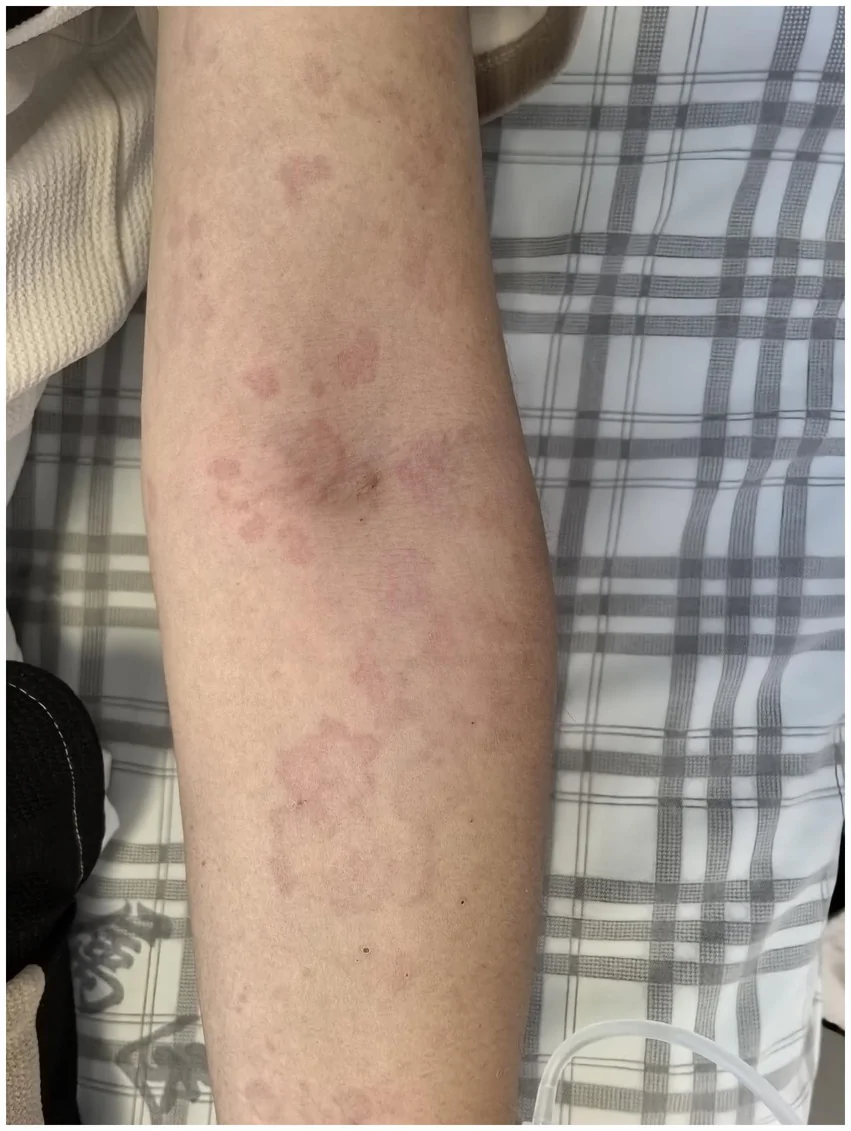
Clinical presentation of skin lesions (upper limb).

**Figure 3:**
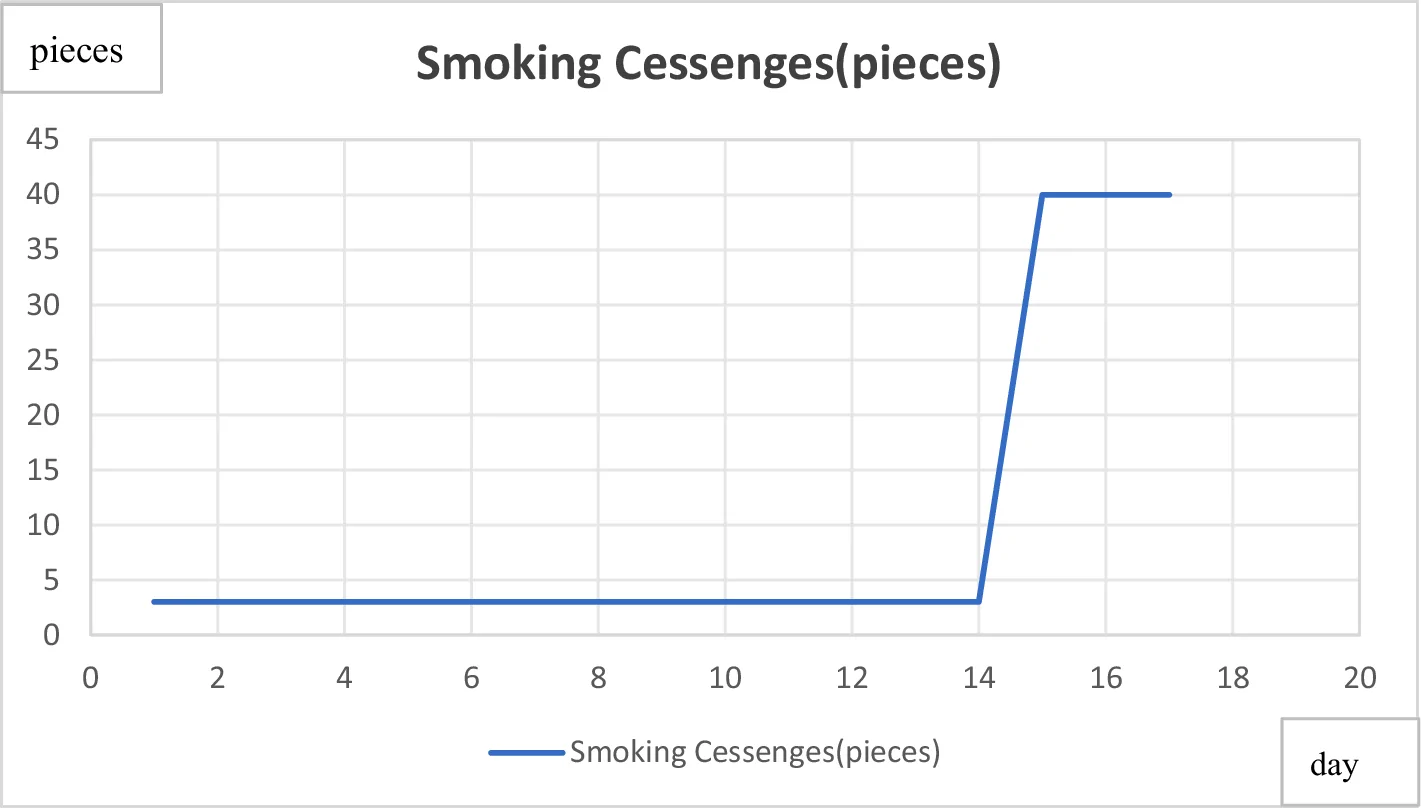
Time and smoking cessenges dose graph.

**Figure 4:**
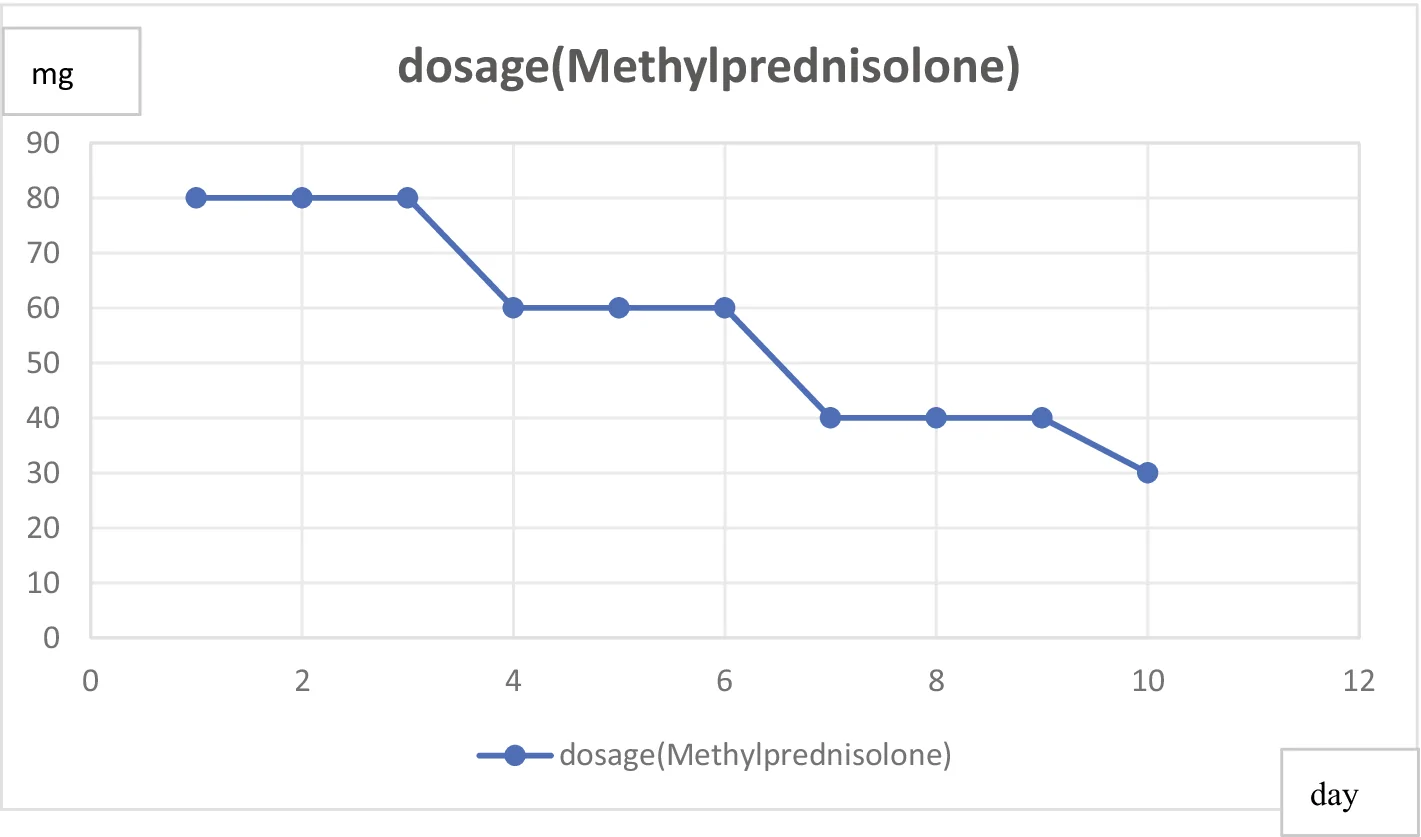
Time and methylprednisolone dose graph.

The patient is a 37-year-old male who presented with widespread red urticaria and pruritus for two days. He reported that he had not taken any medications in the past three months, except for self-purchased smoking cessation lozenges (containing nicotine), which he has been taking for two weeks at a rate of approximately three pieces daily. Recently, the patient has not been working and has been playing mahjong. With nothing to do in his free time, he has taken a large amount of smoking cessation candies. In the last three days, the patient increased his intake to about 40 pieces of smoking cessation lozenges per day. On examination, the patient exhibited extensive urticaria, accompanied by abdominal pain and respiratory distress (Figures 1, 2). He described the itching as significantly bothersome. Laboratory tests revealed a white blood cell count of 10.66 × 10^9^/L, with neutrophils at 8.62 × 10^9^/L. The patient’s procalcitonin (PCT) and C-reactive protein levels were not found to be abnormally high, and the throat swab test results were also normal. The humoral immunity, autoimmunity antibody profile, antinuclear antibody, and infection markers (such as hepatitis B, hepatitis C, syphilis, HIV, etc.) were all within normal ranges. No abnormalities were found in liver function and kidney function. Additionally, interleukin-6 levels were measured at 154.93 pg/mL. An abdominal CT scan indicated the presence of multiple lymph nodes in the bilateral pelvic and inguinal regions, with some enlargement observed in the left pelvic wall lymph nodes. The patient has no history of allergies, nor any history of atopic diseases such as asthma, eczema or allergic rhinitis. The patient has a smoking history (smoking for 10 years, 20 cigarettes per day), and no regular medication was taken before the attack. This was the first occurrence of urticaria. The patient has no fever, and throat swab and chest CT examinations show no obvious signs of infection. There has been no recent consumption of unusual foods, no medication use, and the patient has been on leave at home recently. The number of pieces of smoking cessation lozenges taken by the patient and the time chart are shown in Figure 3. The clinical presentation of widespread urticaria led to a differential diagnosis centered around an allergic reaction, potentially induced by excessive nicotine intake from the smoking cessation lozenges. The significant increase in nicotine consumption in the preceding days likely contributed to the acute onset of symptoms. The patient was treated with intravenous administration of methylprednisolone sodium succinate at a dosage of 40 mg twice daily for anti-inflammatory effects. Additionally, pantoprazole at 40 mg once daily was given intravenously to protect the gastric mucosa. Levofloxacin was administered for infection prophylaxis for three days, and nebulized treatments included a combination of terbutaline and dexamethasone suspension. Antihistamines, including desloratadine and ebastine, were also provided for symptomatic relief of itching. After treatment with 80 mg of methylprednisolone for three days, the patient’s red urticaria and pruritus decreased compared with those at admission, and the dyspnea and abdominal pain resolved. The methylprednisolone dose was then reduced to 60 mg daily. After three more days of treatment, the patient’s red urticaria and pruritus were significantly reduced, with no new skin lesions observed. The methylprednisolone dose was further reduced to 40 mg daily. After another three days of treatment, the patient’s red urticaria and pruritus resolved completely, and no new skin lesions were seen. Treatment with 30 mg of methylprednisolone for one day continued to show no new skin lesions (Figure 4). Following ten days of treatment, the patient’s condition improved significantly, leading to his discharge from the hospital.

## Discussion

This report presents a case of acute urticaria with a suspected, though not established, connection to excessive nicotine lozenge use. The patient had consumed a large amount of smoking cessation lozenges (40 pieces) 3 days before the onset of symptoms, which was suspected to be an allergic reaction caused by nicotine overdose. Smoking cessation lozenges contains 2–4 mg of nicotine per piece. Upon oral administration, nicotine in its neutral form crosses lipid-based barriers, including cellular membranes, to enter the intracellular compartment. It is quickly transported to the brain, where it easily penetrates the blood–brain barrier and produces its effects (11). Previous studies have highlighted the incidence of acute urticaria stemming from various triggers, including drugs, infections, and environmental factors, with idiopathic origins comprising a significant proportion of cases (12, 13). Notably, while drug-induced urticaria is well-documented, reports specifically associating nicotine with hypersensitivity reactions remain sparse. Lee et al. once reported a case of generalized pruritus and urticaria induced by inhalation of nicotine from tobacco (2). Bircher et al. found that nicotine can cause contact dermatitis (14). A patient developed urticaria after using e-cigarettes, and the urticaria may be associated with e-cigarette use (1).

Furthermore, the case highlights distinctive clinical presentations compared to other documented instances of urticaria. For example, while many cases of acute urticaria are idiopathic, this case presents a possible linkage to excessive nicotine intake, underscoring a possible threshold effect that merits further investigation. Consequently, this case underscores the need for heightened awareness and further research into nicotine as a potential allergen in susceptible individuals.

The acute urticaria observed in this case, may induced by nicotine overdose, raises important considerations regarding the underlying pathophysiological mechanisms and potential diagnostic pitfalls associated with such hypersensitivity reactions. Nicotine, a well-known alkaloid, exerts various pharmacological effects that may lead to hypersensitivity in susceptible individuals. Nicotine may act as a hapten, modifying proteins and thereby eliciting immune responses. The threshold dosage for nicotine-induced urticaria remains unclear, necessitating further research to establish safe consumption levels for smoking cessation products.

The rarity of nicotine-induced urticaria may hinder comprehensive understanding and reporting of similar cases in the literature. Additionally, the unclear threshold dosage for triggering association reactions calls for more extensive research to delineate safe consumption levels for nicotine replacement products. Future studies should focus on elucidating the underlying immunological mechanisms associated with nicotine. Ultimately, this case contributes to the growing body of evidence advocating for an informed and cautious approach to nicotine consumption.

In our study, we did not perform allergy testing, challenge tests, or objective assessments of nicotine exposure. However, we based the diagnosis on a thorough clinical history, including a clear temporal relationship between nicotine exposure and the onset of urticaria, along with the exclusion of other common causes through history and basic laboratory tests.

## Conclusion

In conclusion, this report highlights a case of acute urticaria in which a possible link to excessive nicotine consumption from cessation lozenges cannot be excluded. It emphasizes the need for patient education on the recommended usage of such products.

## Data Availability

The original contributions presented in the study are included in the article/supplementary material, further inquiries can be directed to the corresponding author.
